# Supplementary material for: TRPV4-Rho GTPase complex structures reveal mechanisms of gating and disease
Source: Nat Commun. 2023 Jun 23;14:3732. doi: 10.1038/s41467-023-39345-0 (PMC10290081; doi:10.1038/s41467-023-39345-0)
Supplement: Supplementary file 1 — Supplementary Information [file 41467_2023_39345_MOESM1_ESM.pdf]

Supplementary Information

TRPV4-Rho GTPase complex structures reveal mechanisms of gating and disease

Do Hoon Kwon[1,†], Feng Zhang[1,†], Brett A. McCray[2], Shasha Feng[3], Meha Kumar[2], Jeremy M. Sullivan[2], Wonpil Im[3], Charlotte J. Sumner[2,4] & Seok-Yong Lee[1,*]

Affiliations:
[1] Department of Biochemistry, Duke University School of Medicine, Durham, North Carolina, 27710, USA.
[2] Department of Neurology, Johns Hopkins University School of Medicine, Baltimore, Maryland, 21205, USA
[3] Departments of Biological Sciences, Chemistry, and Bioengineering, Lehigh University, Bethlehem, Pennsylvania, 18015, USA.
[4] Department of Neuroscience, Johns Hopkins University School of Medicine, Baltimore, Maryland, 21205, USA

† These authors contributed equally.

*Correspondence to:
S.-Y. Lee
Email: seok-yong.lee@duke.edu
Telephone: 919-684-1005

Supplementary Information

A. Supporting Information Figures S1-S11 2-19
B. Supporting Information Table S1 20
C. Source gel data. 21-22

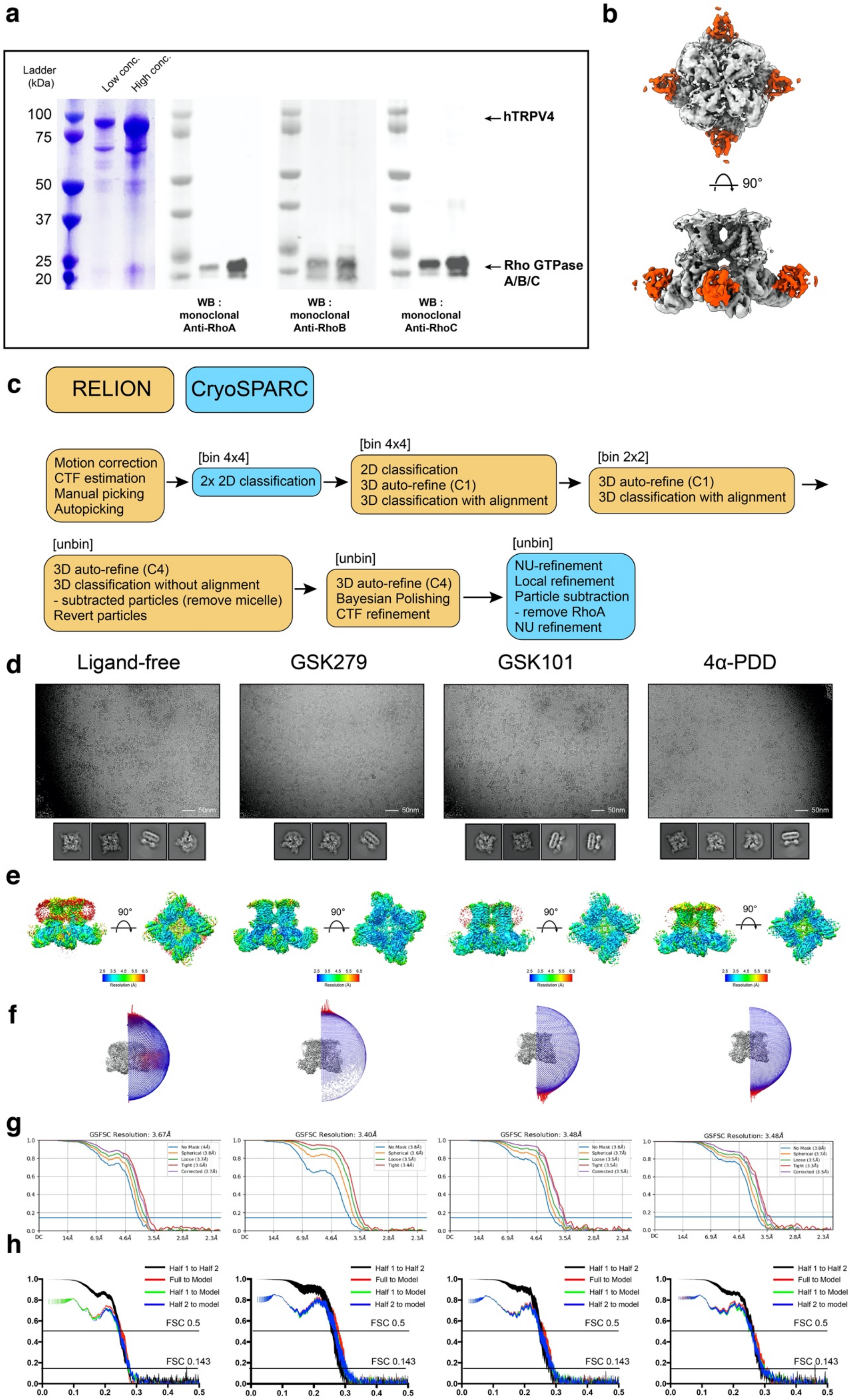

a
Ladder (kDa)
Low conc.
High conc.
100
75
50
37
25
20
WB : monoclonal Anti-RhoA
WB : monoclonal Anti-RhoB
WB : monoclonal Anti-RhoC
hTRPV4
Rho GTPase A/B/C
b
90°
c
RELION
CryoSPARC
Motion correction
CTF estimation
Manual picking
Autopicking
[bin 4x4]
2x 2D classification
[bin 4x4]
2D classification
3D auto-refine (C1)
3D classification with alignment
[bin 2x2]
3D auto-refine (C1)
3D classification with alignment
[unbin]
3D auto-refine (C4)
3D classification without alignment
- subtracted particles (remove micelle)
Revert particles
[unbin]
3D auto-refine (C4)
Bayesian Polishing
CTF refinement
[unbin]
NU-refinement
Local refinement
Particle subtraction
- remove RhoA
NU refinement
d
Ligand-free
GSK279
GSK101
4α-PDD
50nm
e
90°
f
g
GSFSC Resolution: 3.67Å
GSFSC Resolution: 3.40Å
GSFSC Resolution: 3.48Å
GSFSC Resolution: 3.48Å
h
Half 1 to Half 2
Full to Model
Half 1 to Model
Half 2 to model
FSC 0.5
FSC 0.143

Supplementary Fig. 1. Cryo-EM data processing summary

a, SDS-PAGE and western blot of purified TRPV4 show co-purified Rho GTPase. The results shown are representatives of $n \geq 6$ purifications and $n \geq 4$ western blots.

b, Cryo-EM reconstruction of GSK279-TRPV4-RhoA with C1 symmetry. The four RhoA densities (red) are equally strong, indicating that RhoA binds to each protomer of TRPV4.

c, General cryo-EM data processing procedure. Tasks performed in the RELION software are colored in orange and those in cryoSPARC in blue.

d, Representative micrographs of TRPV4-RhoA sample in vitreous ice and 2D classification images. Approximately 1 million particles of each dataset were selected and used for further 3D classifications.

e, Local resolution estimations.

f, Euler distribution plots.

g, Fourier shell correlation (FSC) curves of the final 3D reconstructions with different masks, as calculated in cryoSPARC.

h, Map-to-model correlation plots for both full and half maps.

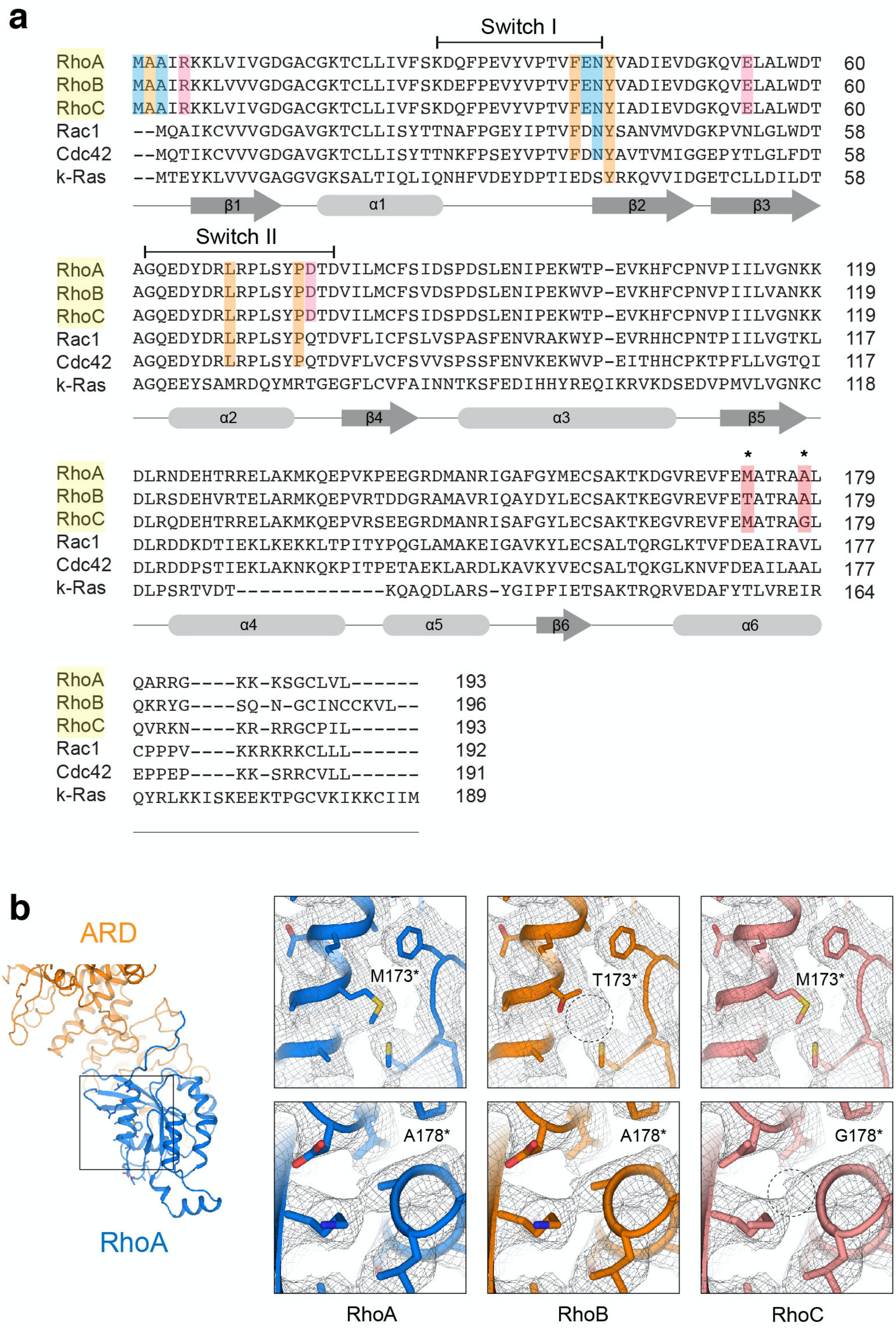
a
Switch I
RhoA MAAIRKKLVIVGDGACGKTCLLIVFSKDQFPEVYVPTVFENYVADIEVDGKQVELALWDT 60
RhoB MAAIRKKLVVVGDGACGKTCLLIVFSKDEFPEVYVPTVFENYVADIEVDGKQVELALWDT 60
RhoC MAAIRKKLVIVGDGACGKTCLLIVFSKDQFPEVYVPTVFENYIADIEVDGKQVELALWDT 60
Rac1 --MQAIKCVVVGDGAVGKTCLLISYTTNAFPGEYIPTVFDNYSANVMVDGKPVNLGLWDT 58
Cdc42 --MQTIKCVVVGDGAVGKTCLLISYTTNKFPSEYVPTVFDNYAVTVMIGGEPYTLGLFDT 58
k-Ras --MTEYKLVVVGAGGVGKSALTIQLIQNHFVDEYDPTIEDSYRKQVVIDGETCLLDILDT 58
β1 α1 β2 β3
Switch II
RhoA AGQEDYDRLRPLSYPDTDVILMCFSIDSPDSLENIPEKWTP-EVKHFCPNVPIILVGNKK 119
RhoB AGQEDYDRLRPLSYPDTDVILMCFSVDSPDSLENIPEKWVP-EVKHFCPNVPIILVANKK 119
RhoC AGQEDYDRLRPLSYPDTDVILMCFSIDSPDSLENIPEKWTP-EVKHFCPNVPIILVGNKK 119
Rac1 AGQEDYDRLRPLSYPQTDVFLICFSLVSPASFENVRAKWYP-EVRHHCPNTPIILVGTKL 117
Cdc42 AGQEDYDRLRPLSYPQTDVFLVCFSVVSPSSFENVKEKWVP-EITHHCPKTPFLLVGTQI 117
k-Ras AGQEEYSAMRDQYMRTGEGFLCVFAINNTKSFEDIHHYREQIKRVKDSEDVPMVLVGNKC 118
α2 β4 α3 β5
* *
RhoA DLRNDEHTRRELAKMKQEPVKPEEGRDMANRIGAFGYMECSAKTKDGVREVFEMATRAAL 179
RhoB DLRSDEHVRTELARMKQEPVRTDDGRAMAVRIQAYDYLECSAKTKEGVREVFETATRAAL 179
RhoC DLRQDEHTRRELAKMKQEPVRSEEGRDMANRISAFGYLECSAKTKEGVREVFEMATRAGL 179
Rac1 DLRDDKDTIEKLKEKKLTPITYPQGLAMAKEIGAVKYLECSALTQRGLKTVFDEAIRAVL 177
Cdc42 DLRDDPSTIEKLAKNKQKPITPETAEKLARDLKAVKYVECSALTQKGLKNVFDEAILAAL 177
k-Ras DLPSRTVDT-------------KQAQDLARS-YGIPFIETSAKTRQRVEDAFYTLVREIR 164
α4 α5 β6 α6
RhoA QARRG----KK-KSGCLVL------ 193
RhoB QKRYG----SQ-N-GCINCCKVL-- 196
RhoC QVRKN----KR-RRGCPIL------ 193
Rac1 CPPPV----KKRKRKCLLL------ 192
Cdc42 EPPEP----KK-SRRCVLL------ 191
k-Ras QYRLKKISKEEKTPGCVKIKKCIIM 189
b
ARD
RhoA
M173*
T173*
M173*
A178*
A178*
G178*
RhoA
RhoB
RhoC

Supplementary Fig. 2. Sequence alignment of the Rho GTPase family

a, Secondary structure elements based on RhoA are shown as gray cylinders (helices) and arrows (beta-strand). The Rho GTPase residues critical for TRPV4 interaction are highlighted: blue for backbone - M1, A3, E40, N41, pink for sidechain - R5, E54, D76, orange for hydrophobic - A2, F39, Y42, L69, P75, and red for subtype-specific sites M173 and A178.

b, Close-up views of the α6-β5 region of Rho GTPase in the TRPV4 ARD-RhoA focused-map. The dotted circles indicate cryo-EM density unaccounted for in the respective models for RhoB and RhoC. Gray mesh indicates cryo-EM densities contoured at 0.15 thresholding.

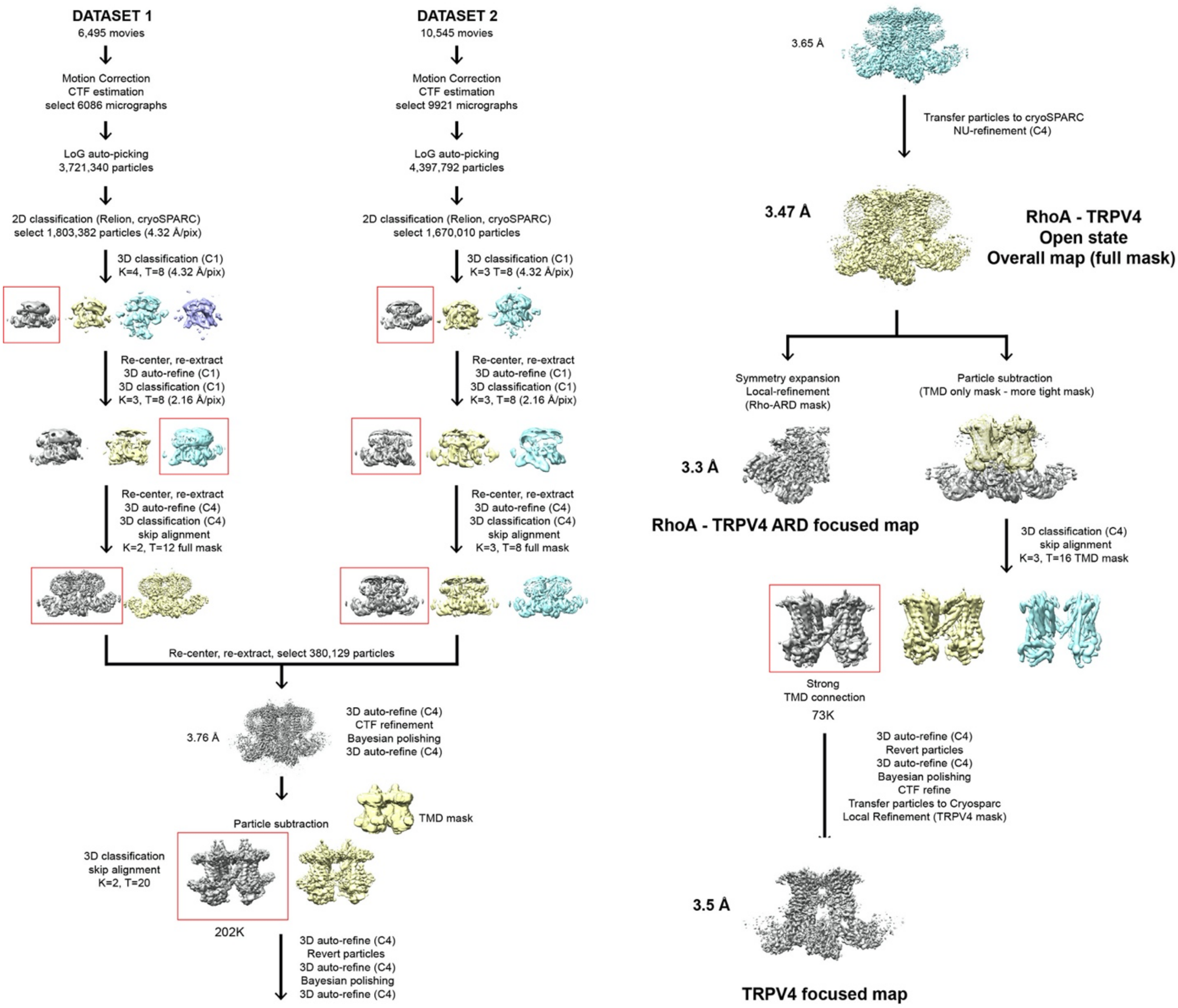

Supplementary Fig. 3. Cryo-EM data processing of the open state (GSK101) structure

The detailed procedure for determining the open state GSK101-TRPV4-RhoA structure.

The final cryo-EM reconstruction was resolved at a resolution of 3.47 Å for GSK101-TRPV4-RhoA, at 3.3 Å for the RhoA-ARD focused map, and at 3.5 Å for the TRPV4 focused map.

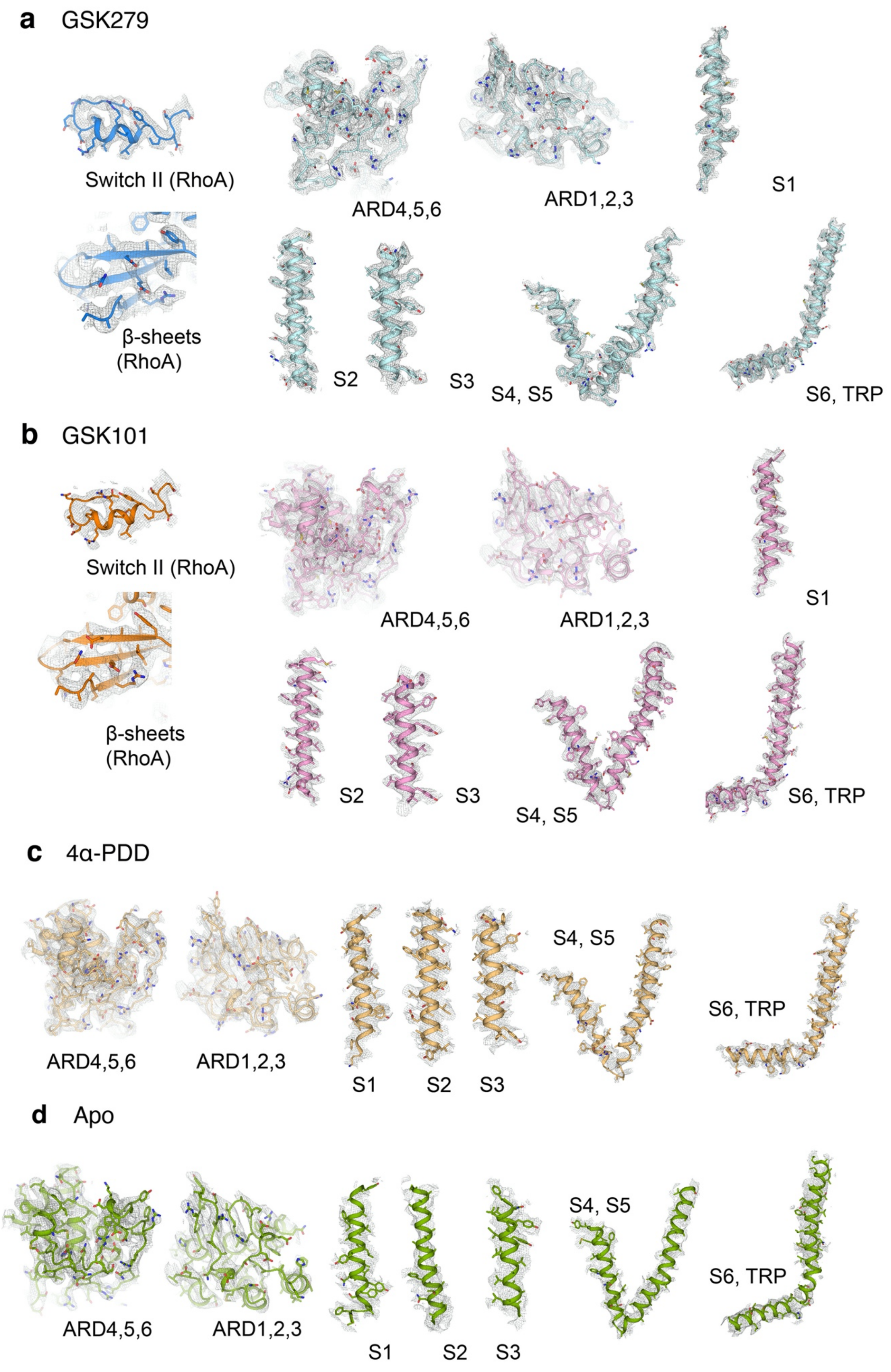

a GSK279
Switch II (RhoA)
ARD4,5,6
ARD1,2,3
S1
β-sheets (RhoA)
S2
S3
S4, S5
S6, TRP
b GSK101
Switch II (RhoA)
ARD4,5,6
ARD1,2,3
S1
β-sheets (RhoA)
S2
S3
S4, S5
S6, TRP
c 4α-PDD
ARD4,5,6
ARD1,2,3
S1
S2
S3
S4, S5
S6, TRP
d Apo
ARD4,5,6
ARD1,2,3
S1
S2
S3
S4, S5
S6, TRP

Supplementary Fig. 4. Representative cryo-EM density of the TRPV4-RhoA reconstructions

a-d, Representative cryo-EM densities for various structural elements in the reconstructions of GSK279-TRPV4-RhoA (**a**, blue and cyan), GSK101-TRPV4-RhoA (**b**, orange and pink), 4α-PDD-TRPV4-RhoA, (**c**, light gold), and ligand-free-TRPV4-RhoA (**d**, green). The RhoA densities in the GSK279- and GSK101-bound reconstructions are from the focused-refined maps. EM densities are shown as gray meshes at thresholding of 0.23-0.25, 0.22–0.24, 0.17–0.19, and 0.16–0.18, respectively, in **a-d**.

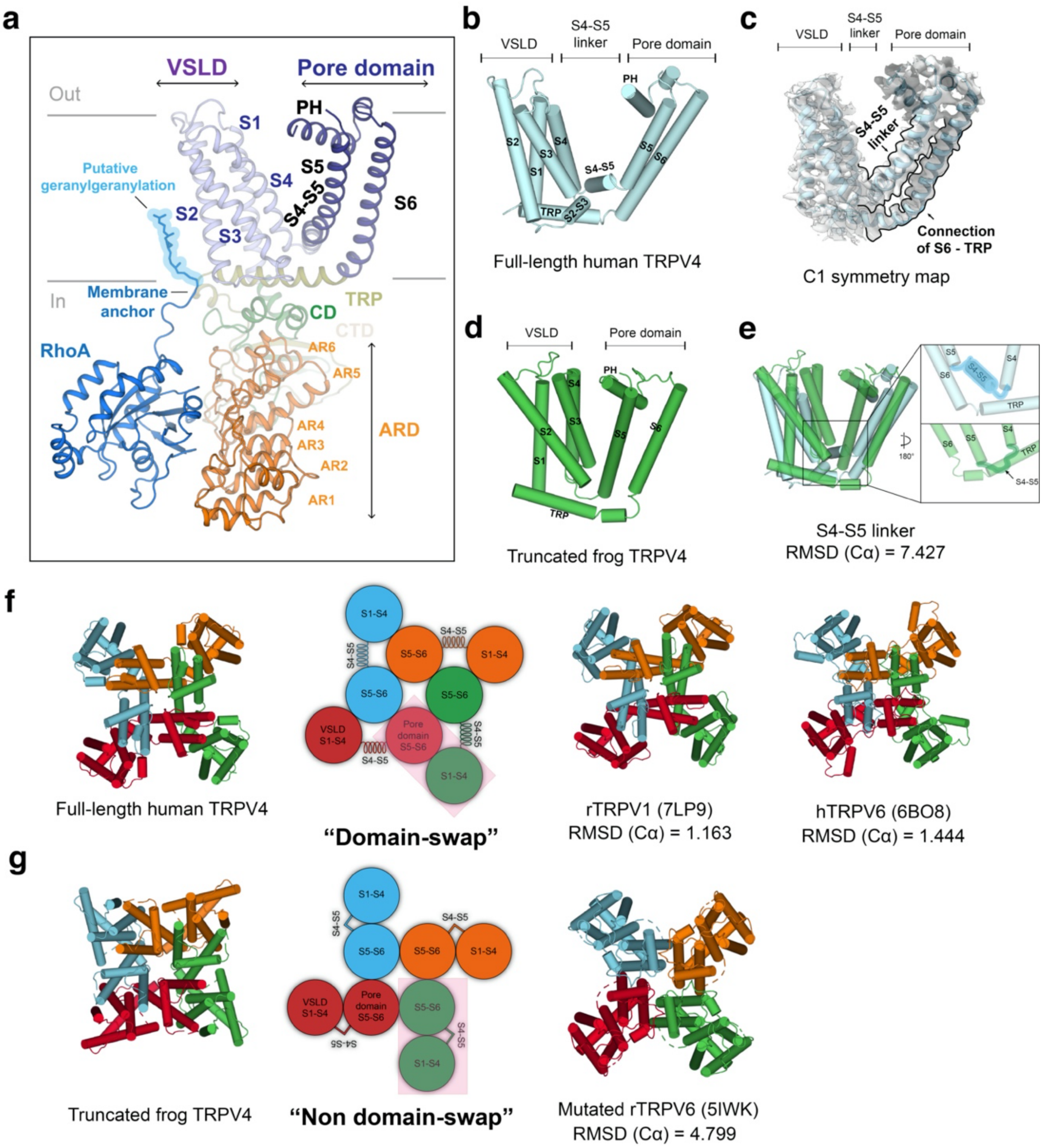

Supplementary Fig. 5. Structural comparisons between human TRPV4 and frog TRPV4

a, Architecture of the human TRPV4 protomer and RhoA with subdomains indicated: ankyrin repeat domain (ARD), coupling domain (CD), transmembrane helices (S1-S6), pore helix (PH), TRP helix, C-terminal domain (CTD), and RhoA. Membrane-anchored lipid tail of RhoA is highlighted in blue.

b, Cylinder representation of the TMD of human TRPV4 in cyan.

c, Cryo-EM density (C1 symmetry) of the GSK279-TRPV4-RhoA complex with the domain-swapping linkers (S4-S5 and S6-TRP) outlined, at thresholding 0.35.

d, Cylinder representation of the TMD of the truncated frog TRPV4 structure in green.

e, Comparison of the TMDs of the human TRPV4 and the frog TRPV4 structures viewed from the membrane plane. The linkers between S4 and S5 are highlighted.

f, The domain-swapping architectures of the human TRPV4, the rat TRPV1, and the human TRPV6 structures.

g, The non-domain-swapping architectures of the truncated frog TRPV4 and the mutated rat TRPV6 structures.

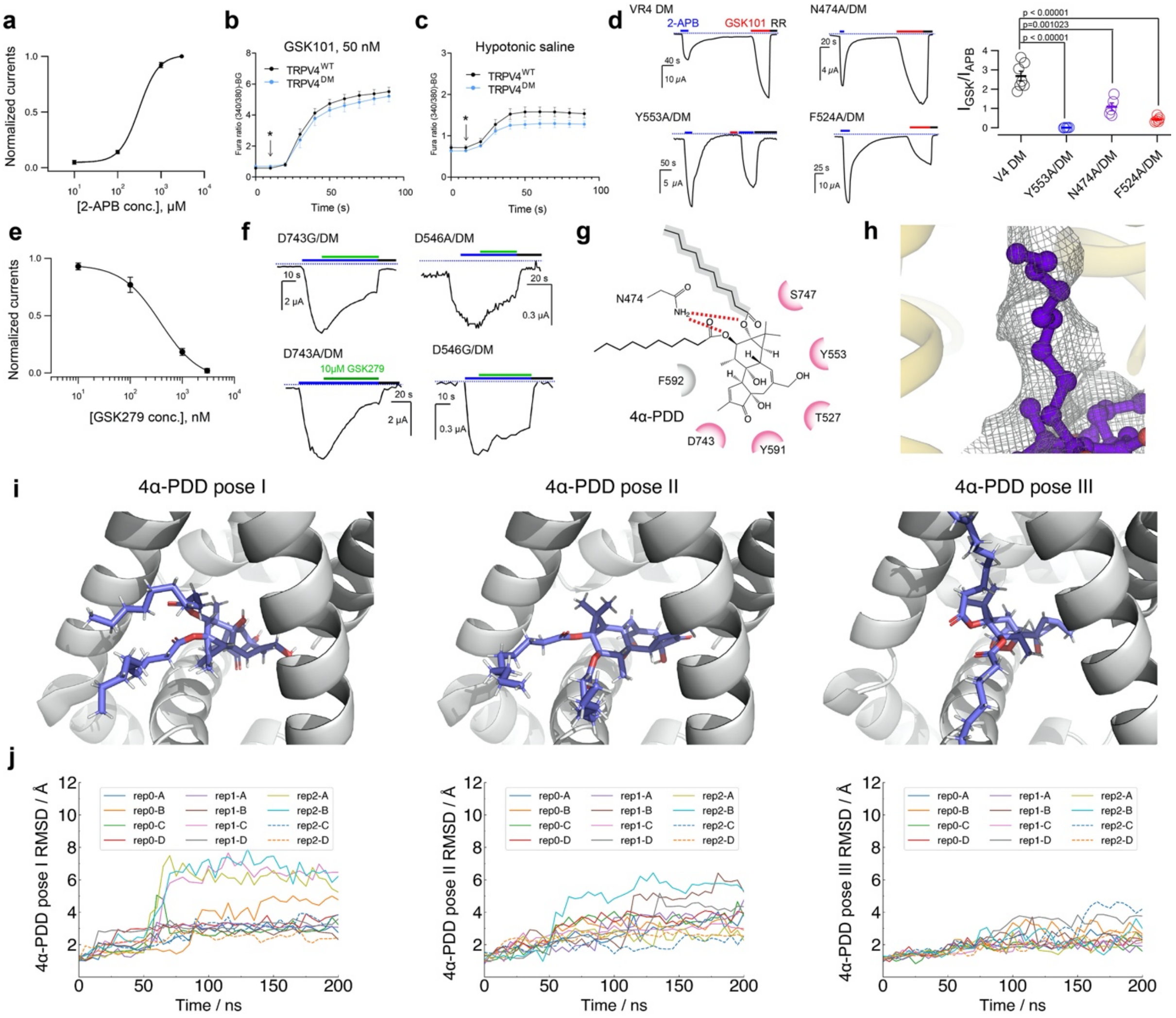

Supplementary Fig. 6. Effects of GSK compounds and osmolarity on TRPV4^{DM} and MD simulation of 4α-PDD binding poses

a, TRPV4^{DM} (VR4^{DM}) mean normalized concentration-response relations for 2-APB. Data are shown as mean ± SEM (n = 4 oocytes). The curves are fit to the Hill equation with EC_{50} = 312 ± 12 µM.

b,c, TRPV4^{DM} mutant shows preserved stimulated calcium influx responses. Averaged ratiometric calcium plots from ratiometric calcium imaging experiments. MN-1 cells were transfected with GFP-tagged TRPV4 plasmids and stimulated with (**b**) GSK101 (50 nM) or (**c**) hypotonic saline. Baseline and hypotonic saline-stimulated calcium responses were then measured over time and then averaged, n = 11 wells per condition, with 20-40 transfected cells per well. Data are shown as mean ± SEM.

d, Probing the GSK101 binding site. Two-electrode voltage-clamp (TEVC) recordings of TRPV4^{DM} (N456H/W737R) and additional mutants made in the background of TRPV4^{DM}, as indicated. Currents at -60 mV were elicited by 2 mM 2-APB, then 5 µM GSK101 was applied,

and 20 μM ruthenium red (RR) was finally introduced, as indicated by colored horizontal lines. (Right) Summary of the ratio between currents activated by 2 mM 2-APB and 5 μM GSK101, data are shown as mean ± SEM, for VR4 DM, Y553A/DM, N474A/DM, F524A/DM, n=7, 5, 5, 6 oocytes, respectively, *P* values are calculated by two-tailed Student's t test as shown in the figure.

e, Concentration-response curve for the effects of GSK279 on the 2 mM 2-APB-stimulated currents as a percent of the maximal inhibition response with the presence of 50 μM RR. The values are expressed as a mean ± SEM. n=4-5 oocytes for each data point, IC_{50}=379.8 ± 32.2 nM.

f, Probing the inhibitor binding site. TEVC recordings of $TRPV4^{DM}$ and additional mutants made in the background of $TRPV4^{DM}$, as indicated. Currents at -60 mV induced by 2 mM 2-APB then co-application of 4 μM GSK279 followed by 20 μM RR, as indicated by colored horizontal lines.

g, Ligplot[94] schematics of the 4α-PDD interaction with TRPV4, with key chemical positions labelled. Pink colored residues are involved in both GSK101 and GSK279 bindings.

h, Cryo-EM densities (gray mesh) for the C-terminal tail of 4α-PDD (violet stick). Densities are contoured at 0.28 thresholding.

i, Illustration of ligand-binding poses of 4α-PDD: pose I (left), pose II (middle), and pose III (right).

j, Ligand RMSD values of 4α-PDD pose I show large deviations from the initial configuration with an average RMSD of 3.53Å (left). Ligand RMSD values of 4α-PDD pose II show large deviations from the initial configuration with an average RMSD of 3.65Å (middle). Ligand RMSD values of 4α-PDD pose III show relatively stable ligand binding with an average RMSD of 2.55 Å, except for one outliner ligand, rep2-D, which stumbles out of the pocket (right). Source data for (**a-f**) are provided as a Source Data file.

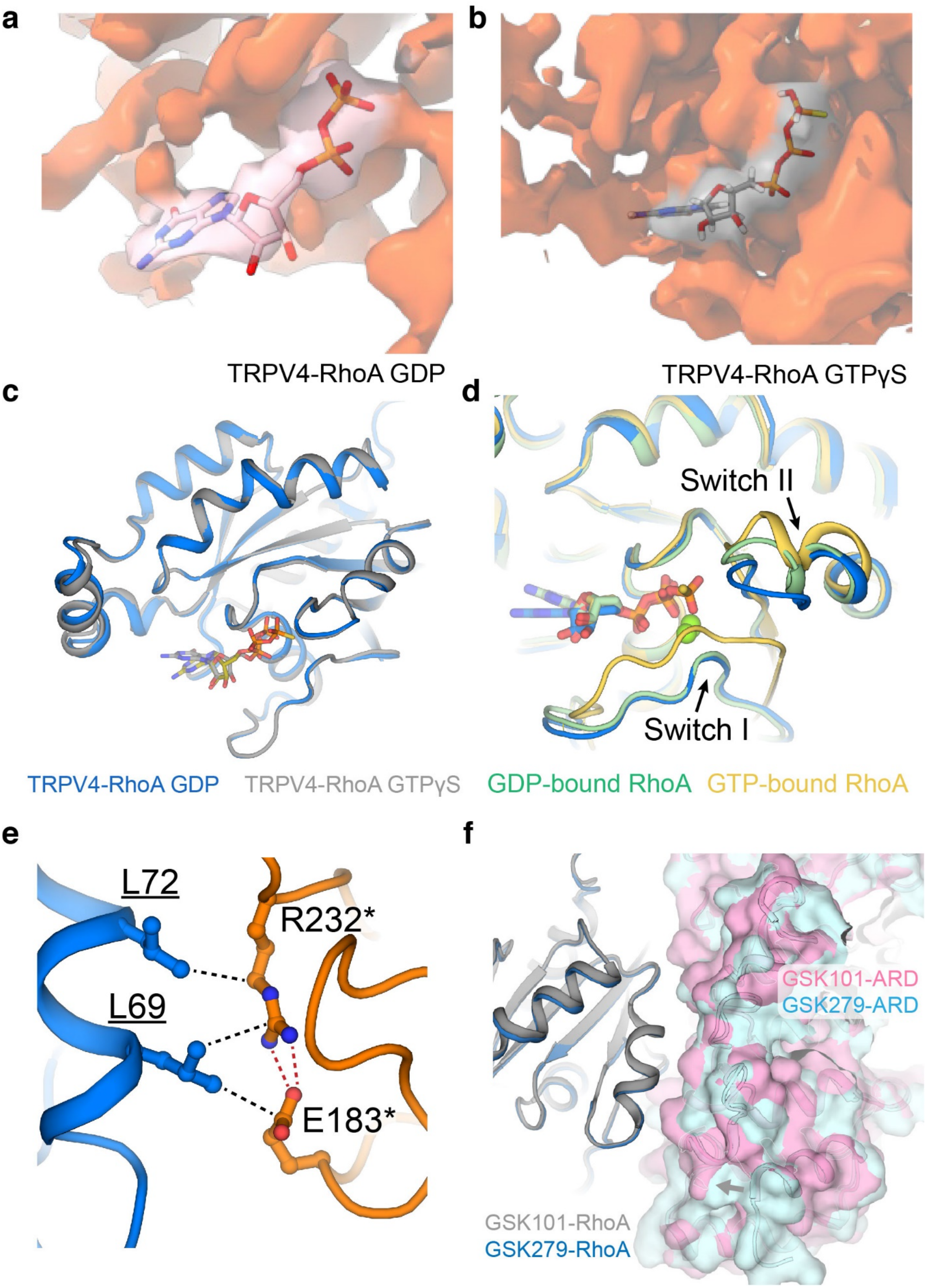

a
b
TRPV4-RhoA GDP
TRPV4-RhoA GTPγS
c
d
Switch II
Switch I
TRPV4-RhoA GDP
TRPV4-RhoA GTPγS
GDP-bound RhoA
GTP-bound RhoA
e
f
L72
L69
R232*
E183*
GSK101-ARD
GSK279-ARD
GSK101-RhoA
GSK279-RhoA

Supplementary Fig. 7. Comparisons of structures of RhoA in complex with TRPV4 and published structures of RhoA alone

a,**b**, Close-up view at the nucleotide binding site of RhoA in 3D reconstructions of GSK279-TRPV4-RhoA-GDP (**a**) and GSK101-TRPV4-RhoA-GTPγS (**b**) at thresholding 0.17 and 0.135, respectively. Nucleotides are shown as sticks.

c, Comparison of the conformations of RhoA in GSK279-TRPV4-RhoA-GDP and GSK101-TRPV4-RhoA-GTPγS. Nucleotides are shown as sticks.

d, Comparison of RhoA conformations in GSK279-TRPV4-RhoA-GDP (blue), GDP-bound RhoA alone (PDB-1FTN, green), and GTPγS-bound RhoA alone (PDB-1A2B, gold). Nucleotides are shown as sticks and magnesium ions are shown as spheres.

e, Interaction of TRPV4-ARD and RhoA. The salt bridge between R232 and E183 in TRPV4 appears to be important for RhoA binding. Underlined residues located in RhoA, and annotated residues indicate disease-causing mutations. The red and black dotted lines indicate salt bridges and hydrophobic interactions, respectively.

f, Comparison of the interaction interface between GSK279-TRPV4-RhoA-GDP and GSK101-TRPV4-RhoA-GTPγS.

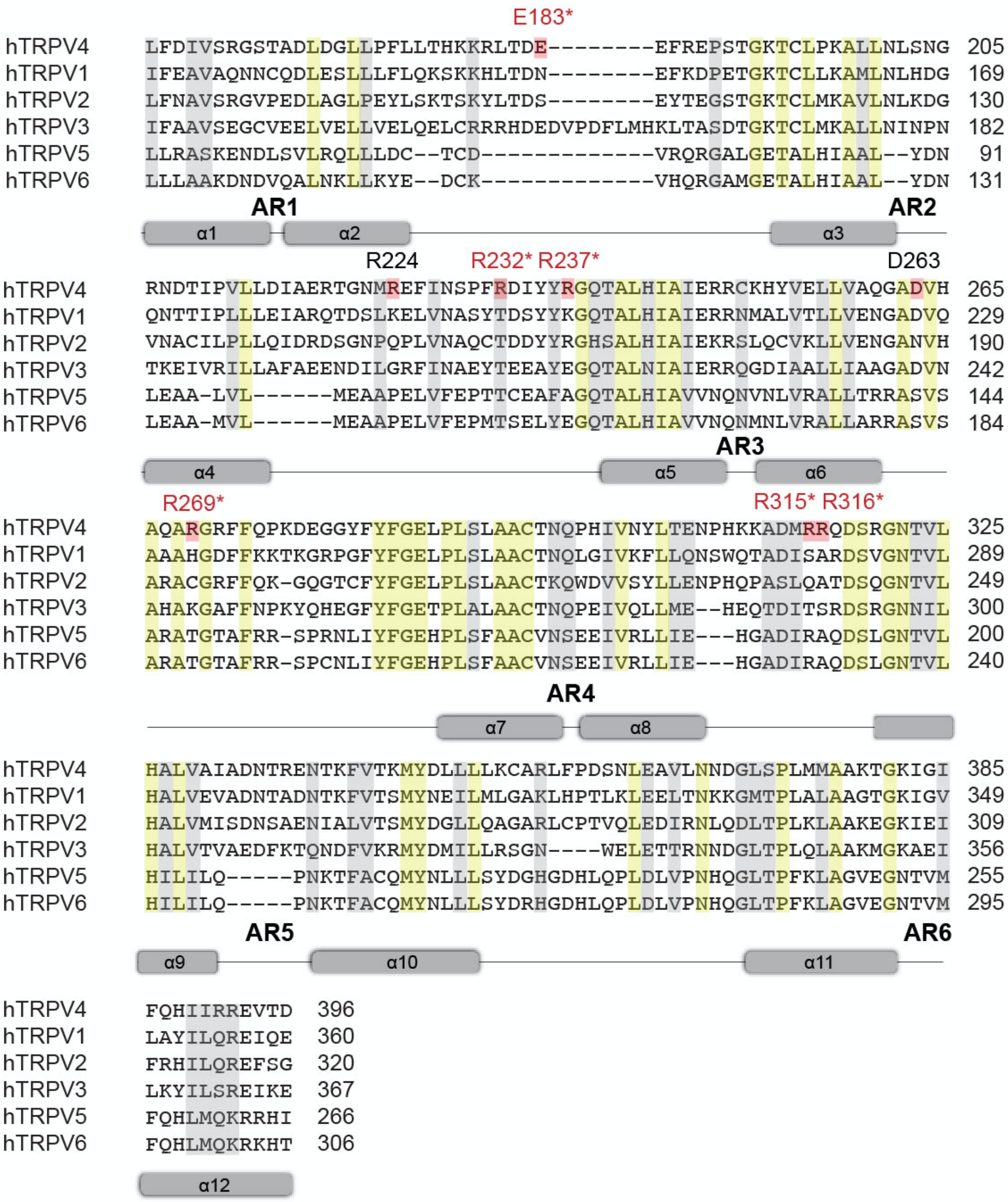

Supplementary Fig. 8. Sequence alignment of the ARDs in human TRPV channels

The α-helices are shown as gray cylinders, well-conserved residues are highlighted in yellow (identical) and gray (similar), and key residues for TRPV4 interaction are colored in red. Asterisks indicate residues mutated in TRPV4-mediated neuromuscular disease.

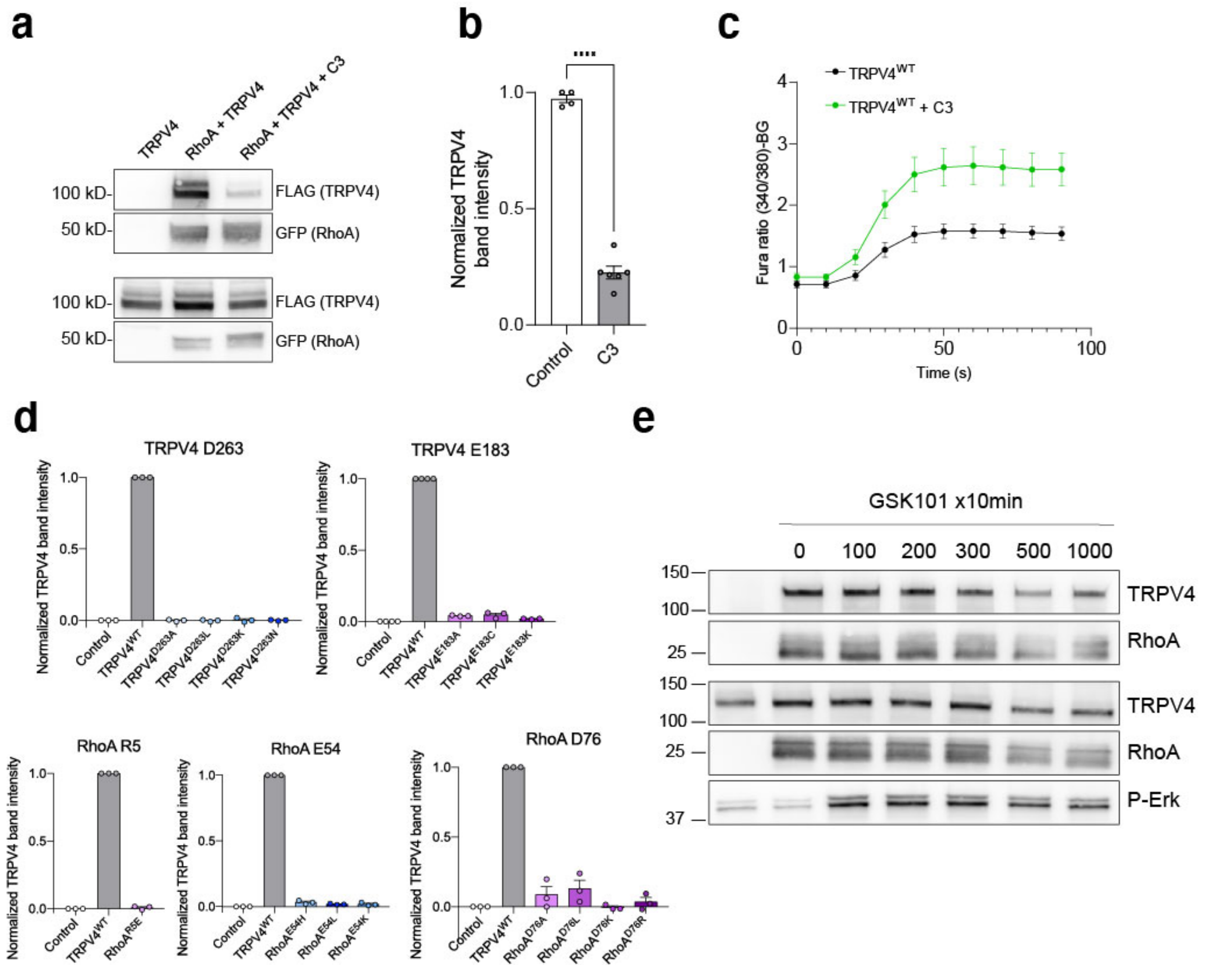

Supplementary Fig. 9 Inhibition of the interaction between TRPV4 and RhoA by RhoA inhibitors and mutations at the interface

a, Co-immunoprecipitation of HEK293T cells transfected with TRPV4-FLAG and RhoA-GFP with or without treatment with the RhoA inhibitor C3 transferase (0.5 µg/ml) for 12 hours demonstrates reduced TRPV4-RhoA interaction.

b, Quantification of densitometry of TRPV4 bands on western blots, n = 4 (control) and 6 (C3 transferase). ****p<0.0001. *P* value was calculated using two-tailed unpaired t test. Data are shown as mean ± SEM.

c, Averaged ratiometric calcium plots from ratiometric calcium imaging experiments. MN-1 cells were transfected with GFP-tagged TRPV4 plasmids and treated with C3 transferase (1 µg/ml) or vehicle for 2 hours and then stimulated with hypotonic saline. Baseline and hypotonic saline-stimulated calcium responses were then measured over time and then averaged, n = 11 wells per condition for control and 12 wells per condition for C3 transferase, with 20-40 transfected cells per well. Data are shown as mean ± SEM.

d, Quantification of densitometry of TRPV4 bands on western blots, n = 3 independent experiments for each set of mutants. Data are shown as mean ± SEM.

e, Representative western blot of co-immunoprecipitation of TRPV4-GFP and RhoA-Myc expressed in HEK293T cells, 1:2 ratio, IP: anti-Myc. Cells were treated with the indicated concentrations (μM) of GSK101 for 10 minutes prior to lysis. The experiment was performed three times with similar results. Data are presented as means ± SEM. Source data for (**b**-**d**) are provided as a Source Data file.

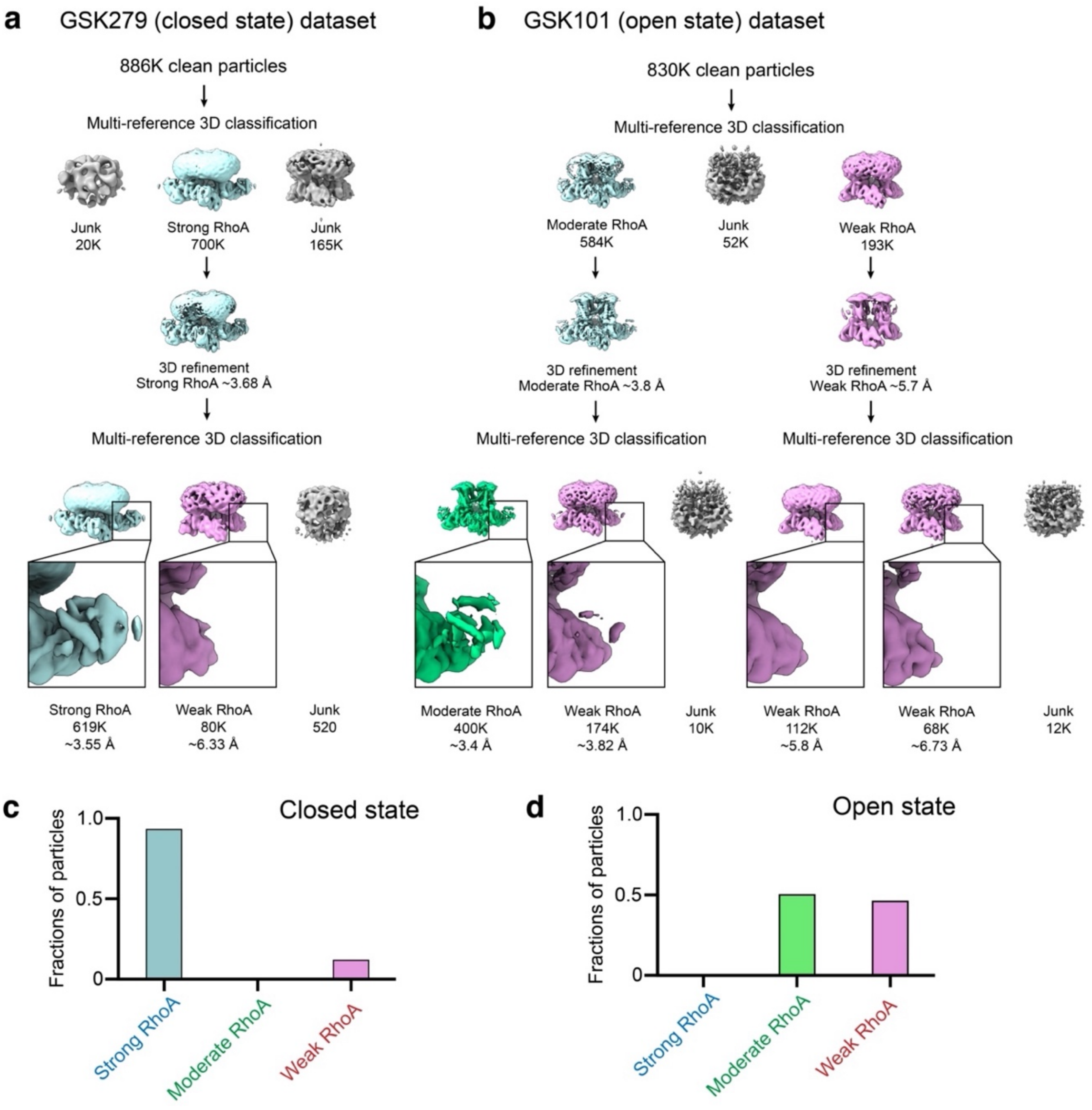

Supplementary Fig. 10. Image processing workflow for the RhoA-bound fraction analysis

a, Data processing workflow of GSK279-TRPV4-RhoA dataset for the purpose of the RhoA-bound fraction analysis. 3D reconstruction thresholding of 0.58.

b, Data processing workflow of GSK101-TRPV4-RhoA dataset for the purpose of the RhoA-bound fraction analysis. The red- and blue-outlined subsets indicate the RhoA-bound fraction and RhoA-unbound fraction, respectively. 3D reconstruction thresholding of 0.58.

c, Class distributions of particles for GSK279-TRPV4-RhoA.

d, Class distributions of particles for GSK101-TRPV4-RhoA.

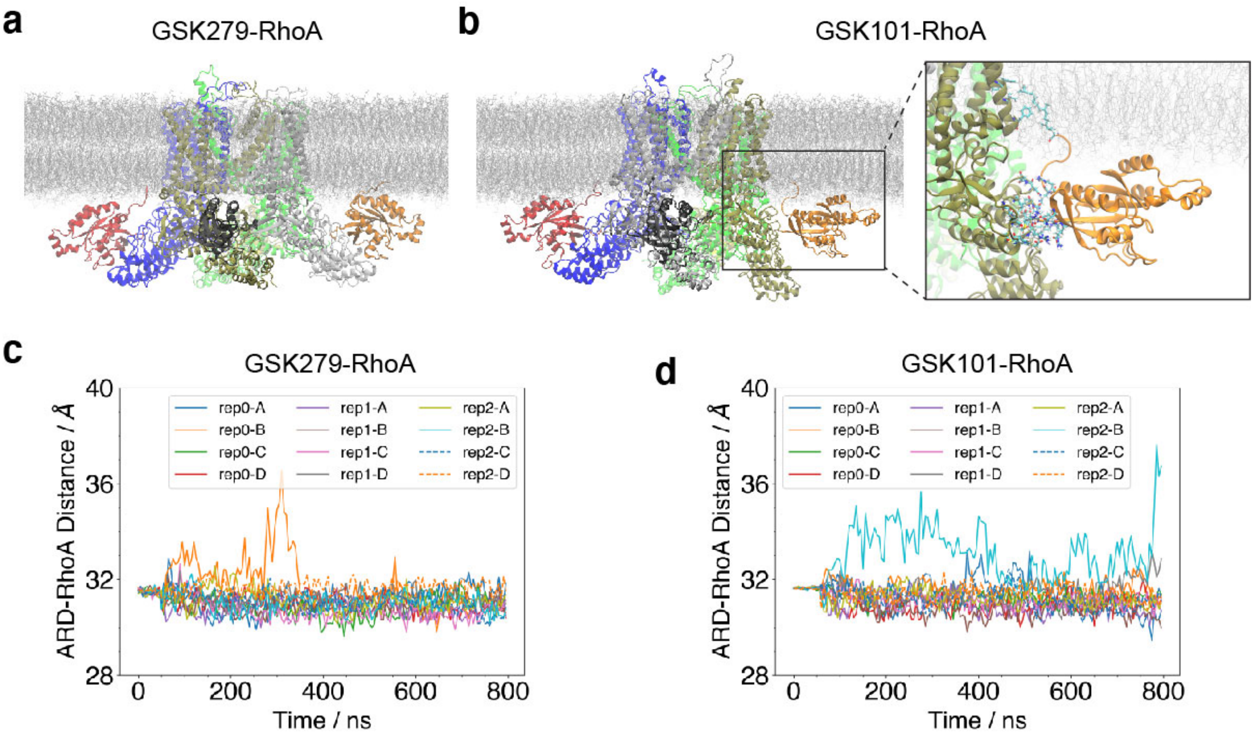

Supplementary Fig. 11. RhoA binding stability to TRPV4 ARD in the MD simulations

a, A representative snapshot at the end of 800-ns simulation of GSK279-TRPV4-RhoA complex. RhoA proteins stay bound to TRPV4.

b, A snapshot at the end of 800-ns simulation of GSK101-TRPV4-RhoA complex where the RhoA protein flips up and loses the majority of the polar contacts with TRPV4. Inset shows the residues that are in contact, a few polar contacts remain in the flip-up configuration. prenylated tail of RhoA interacts with hydrophobic residues of S2 helix.

c,**d**, Center-of-mass distance between RhoA and TRPV4 ARD as a function of time for both GSK279-bound (c) and GSK101-bound (d) states. In the case of GSK101-bound, one replica site shows persistent RhoA fluctuations and ends up in the flip-up pose.

Supplementary Table 1. Cryo-EM data collection, refinement, and validation statistics.

	TRPV4-RhoA GSK2798745 (EMDB-28975) (PDB-8FC7)	TRPV4 GSK1016790A (EMDB-28976) (PDB-8FC8)	TRPV4-RhoA GSK1016790A (PDB-8FCB)	TRPV4-RhoA ligand-free (EMDB-28977) (PDB-8FC9)	TRPV4 4α-PDD (EMDB-28978) (PDB-8FCA)
**Data collection and processing**					
Magnification	81,000	81,000		81,000	81,000
Voltage (kV)	300	300		300	300
Electron exposure (e^-/Å^2)	60	60		60	60
Defocus range (μm)	-0.8 to -1.8	-0.8 to -1.8		-0.8 to -1.8	-0.8 to -1.8
Pixel size (Å)	1.08	1.08		1.08	1.08
Symmetry imposed	C4	C4		C4	C4
Initial particles images (no.)	2,111,563	8,119,132		3,412,804	4,418,471
Final particles images (no.)	119,462	73,349	207,645	172,225	244,007
Map resolution (Å)	3.3	3.52	3.43	3.75	3.45
FSC threshold	0.143	0.143	0.143	0.143	0.143
**Refinement**					
Initial model used (PDB code)	TRPV1 (7LP9) RhoA (1FTN)	PDB-8FC7	PDB-8FC7	PDB-8FC7	PDB-8FC7
Map sharpening *B* factor (Å^2)	-136	-160	-183	-195	-165
Model composition					
Non-hydrogen atoms	25,716	19,768	25,232	21,812	18,460
Protein residues	3,284	2,468	3,228	3,092	2,400
Ligands	12	8	12	0	8
B factors(Å^2)					
Protein	89.95	82.56	101.89	136.85	91.41
Ligand	68.98	50.97	44.81	-	62.40
R.m.s deviations					
Bond lengths (Å)	0.004	0.003	0.004	0.004	0.005
Bond angles (°)	1.077	0.568	0.967	0.562	0.649
**Validation**					
MolProbity score	1.58	1.17	1.39	1.06	1.33
Clashscore	5.47	2.35	4.14	3.81	6.01
Poor rotamers (%)	0	0.19	0.16	0.22	0
Ramachandran plot					
Favored (%)	95.83	97.15	96.85	98.56	98.32
Allowed (%)	4.17	2.85	3.15	1.44	1.68
Disallowed (%)	0	0	0	0	0

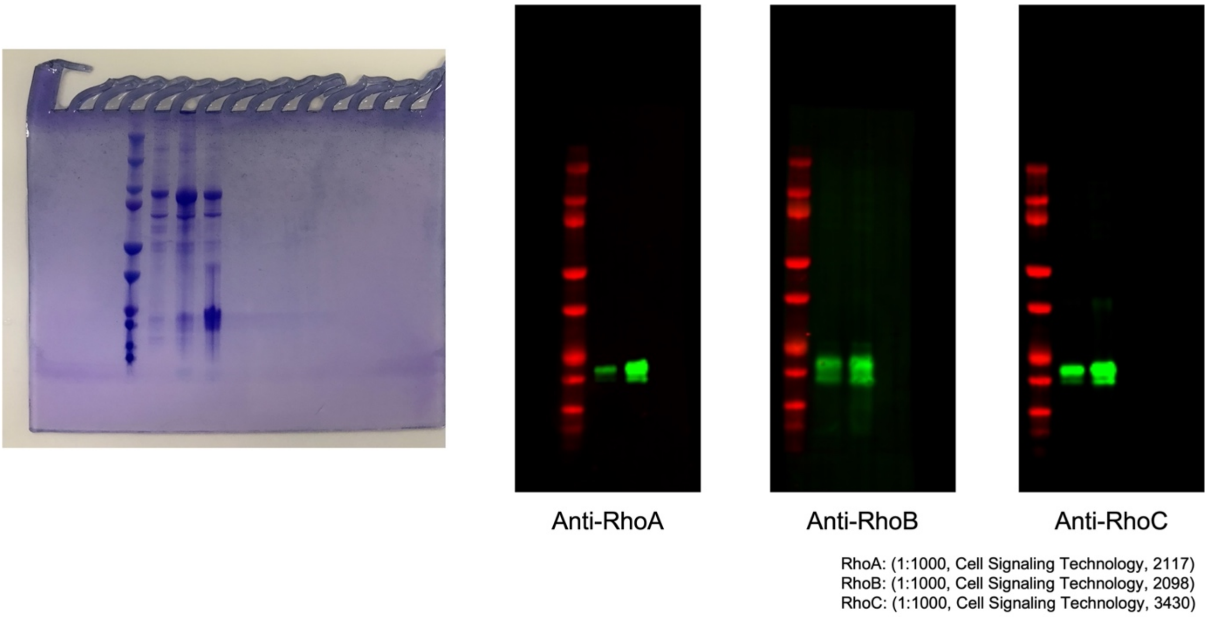

Source Data for Supplementary Fig. 1a.

The uncropped gel and blots used in Supplementary Fig. 1a are shown.

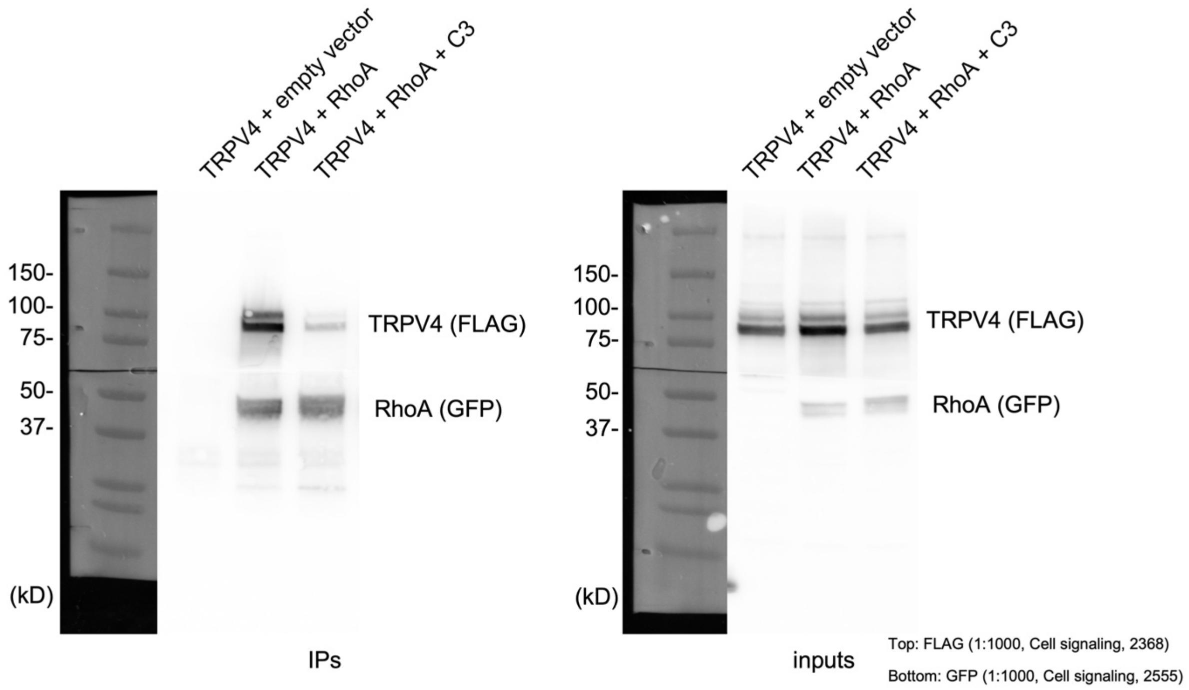

Source Data for Supplementary Fig. 9a.

The uncropped blots used in Supplementary Fig. 9a are shown.

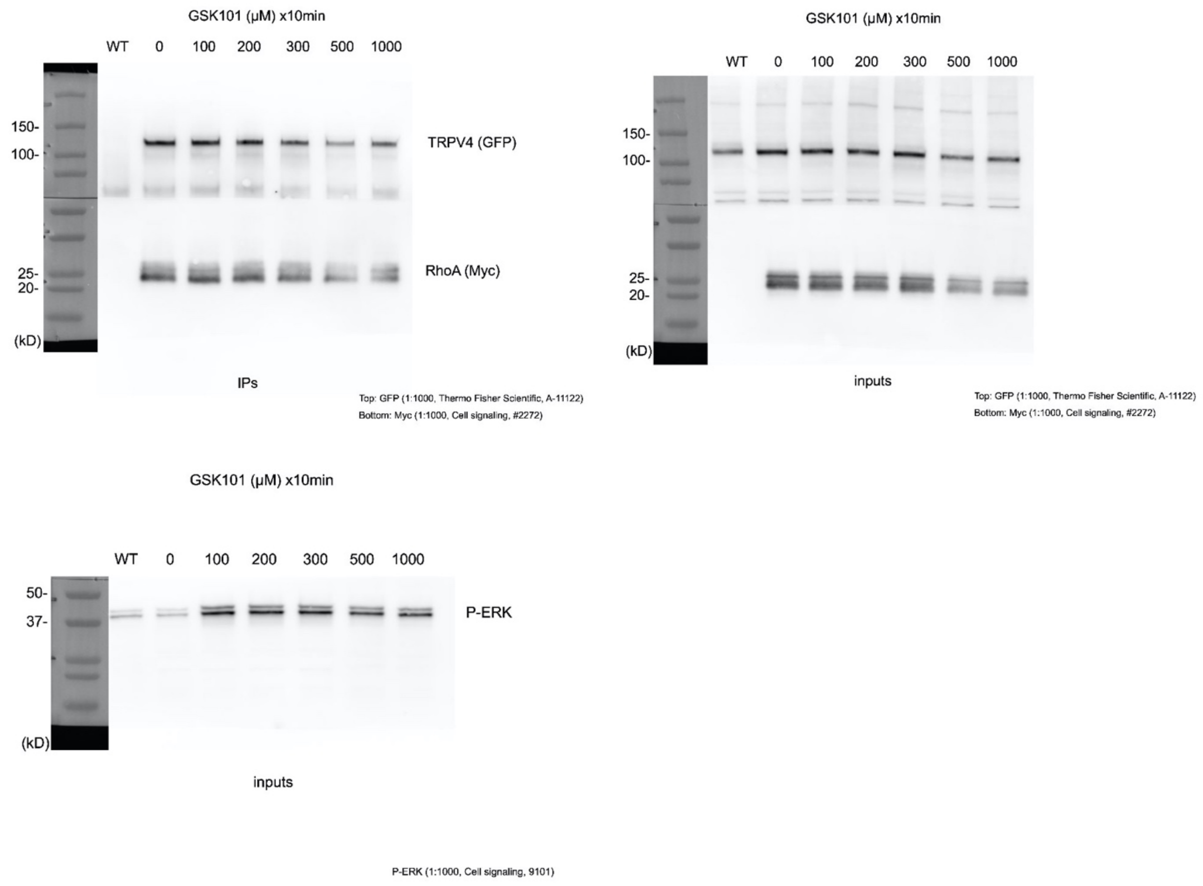

Source Data for Supplementary Fig. 9e.

The uncropped blots used in Supplementary Fig. 9e are shown.
