# Supplementary material for: TRPV4-Rho GTPase complex structures reveal mechanisms of gating and disease
Source: Nat Commun. 2023 Jun 23;14:3732. doi: 10.1038/s41467-023-39345-0 (PMC10290081; doi:10.1038/s41467-023-39345-0)
Supplement: Supplementary file 3 — Reporting Summary [file 41467_2023_39345_MOESM3_ESM.pdf]

nature portfolio

Corresponding author(s): Seok-Yong Lee

Last updated by author(s): Jun 2, 2023

Reporting Summary

Nature Portfolio wishes to improve the reproducibility of the work that we publish. This form provides structure for consistency and transparency in reporting. For further information on Nature Portfolio policies, see our Editorial Policies and the Editorial Policy Checklist.

Statistics

For all statistical analyses, confirm that the following items are present in the figure legend, table legend, main text, or Methods section.

n/a	Confirmed	
☐	☒	The exact sample size (*n*) for each experimental group/condition, given as a discrete number and unit of measurement
☐	☒	A statement on whether measurements were taken from distinct samples or whether the same sample was measured repeatedly
☐	☒	The statistical test(s) used AND whether they are one- or two-sided<br>*Only common tests should be described solely by name; describe more complex techniques in the Methods section.*
☒	☐	A description of all covariates tested
☐	☒	A description of any assumptions or corrections, such as tests of normality and adjustment for multiple comparisons
☐	☒	A full description of the statistical parameters including central tendency (e.g. means) or other basic estimates (e.g. regression coefficient) AND variation (e.g. standard deviation) or associated estimates of uncertainty (e.g. confidence intervals)
☐	☒	For null hypothesis testing, the test statistic (e.g. *F*, *t*, *r*) with confidence intervals, effect sizes, degrees of freedom and *P* value noted<br>*Give P values as exact values whenever suitable.*
☒	☐	For Bayesian analysis, information on the choice of priors and Markov chain Monte Carlo settings
☒	☐	For hierarchical and complex designs, identification of the appropriate level for tests and full reporting of outcomes
☒	☐	Estimates of effect sizes (e.g. Cohen's *d*, Pearson's *r*), indicating how they were calculated

Our web collection on statistics for biologists contains articles on many of the points above.

Software and code

Policy information about availability of computer code

Data collection	Cryo-EM data was collected using Latitude S 1.3, Ratiometric calcium imaging data was collected using AxioVision software (Zeiss, v4)
Data analysis	Gctf 1.06 -published and freely available, MotionCor2 1.2.6 - published and freely available, Relion 3.1/4.0 - published and freely available, CryoSPARC 3.0/3.1 - published and freely available, Coot 0.8 - published and freely available, PHENIX 1.18 - published and freely available, UCSF Chimera 1.16 - published and freely available, PyMOL 2.5.2 - published and freely available, ChimeraX 1.2.5 - published and freely available, Prism 9 - commercially available, pClamp 10.5 - commercially available

For manuscripts utilizing custom algorithms or software that are central to the research but not yet described in published literature, software must be made available to editors and reviewers. We strongly encourage code deposition in a community repository (e.g. GitHub). See the Nature Portfolio guidelines for submitting code & software for further information.

Data

Policy information about availability of data

All manuscripts must include a data availability statement. This statement should provide the following information, where applicable:

- Accession codes, unique identifiers, or web links for publicly available datasets
- A description of any restrictions on data availability
- For clinical datasets or third party data, please ensure that the statement adheres to our policy

All data supporting the findings of this study and unique biological materials used in this study are available from the corresponding author upon reasonable

request. Coordinates have been deposited in the Protein Data Bank with the PDB IDs - 8FC9 (human TRPV4-RhoA, ligand-free), 8FC7 (human TRPV4-RhoA, GSK279-bound closed), 8FCB (human TRPV4-RhoA, GSK101-bound, open), 8FC8 (human TRPV4 only, GSK101-bound, open), 8FCA (human TRPV4, 4a-PDD-bound, putative open) respectively. The cryo-EM maps have been deposited in the Electron Microscopy Data Bank with the IDs EMD - 28977 (human TRPV4-RhoA, ligand-free), 28975 (human TRPV4-RhoA, GSK279-bound closed), 29030 (human TRPV4-RhoA, GSK279-bound closed, TRPV4-focused), 29031 (human TRPV4-RhoA, GSK279-bound closed, ARD-RhoA focused), 28976 (human TRPV4-RhoA and TRPV4 only, GSK101 bound, open), 29331 (human TRPV4-RhoA and TRPV4 only, GSK101 bound, open, TRPV4-focused), 29332 (human TRPV4-RhoA and TRPV4 only, GSK101 bound, open, ARD-RhoA focused), 28978 (human TRPV4, 4a-PDD-bound, putative open), respectively. The MD simulation data generated in this study have been deposited in the Zenodo OpenAIRE database under accession code 7996190 (https://zenodo.org/record/7996190). We have used the following published structures for the initial model building: 7LP9. Source data are provided with this paper.

Human research participants

Policy information about studies involving human research participants and Sex and Gender in Research.

Reporting on sex and gender	N/A
Population characteristics	N/A
Recruitment	N/A
Ethics oversight	N/A

Note that full information on the approval of the study protocol must also be provided in the manuscript.

Field-specific reporting

Please select the one below that is the best fit for your research. If you are not sure, read the appropriate sections before making your selection.

☒ Life sciences ☐ Behavioural & social sciences ☐ Ecological, evolutionary & environmental sciences

For a reference copy of the document with all sections, see nature.com/documents/nr-reporting-summary-flat.pdf

Life sciences study design

All studies must disclose on these points even when the disclosure is negative.

Sample size	Batch sizes per replicate were determined based on signal-to-noise optimization, and replicates were accumulated to ensure accurate determination of data values. Statistical calculations were used to determine mean and standard deviation and standard error of the mean. Sample sizes required were not calculated.
Data exclusions	No data were excluded.
Replication	All biochemical assays were repeated at least in triplicate ($n \geq 3$) and were reproducible.
Randomization	Samples (cells or oocytes) were not randomized for this study.<br>For each electro-physiology experiment, we need to choose the best behave cell (least leak current, normal cell shape, etc) to record to minimize the background currents (leak currents, for example)<br>For ratiometric calcium imaging, the center of each coverslip was imaged. Experimental conditions were assigned by transfecting successive wells of a 96 well plate with each mutant form of TRPV4, and then repeating the same order to fill the plate.
Blinding	Blinding is not used in our studies since our experiments were to describe the phenomenon that how different mutants channels' react to a compound.<br>Blinding for calcium imaging was deemed unnecessary as generation of cell ROIs and subsequent quantification were performed in a standardized manner with most steps being automated.

Reporting for specific materials, systems and methods

We require information from authors about some types of materials, experimental systems and methods used in many studies. Here, indicate whether each material, system or method listed is relevant to your study. If you are not sure if a list item applies to your research, read the appropriate section before selecting a response.

Materials & experimental systems

n/a	Involved in the study
☐	☒ Antibodies
☐	☒ Eukaryotic cell lines
☒	☐ Palaeontology and archaeology
☒	☐ Animals and other organisms
☒	☐ Clinical data
☒	☐ Dual use research of concern

Methods

n/a	Involved in the study
☒	☐ ChIP-seq
☒	☐ Flow cytometry
☒	☐ MRI-based neuroimaging

Antibodies

Antibodies used

Primary antibodies used were rabbit anti-Myc (Cell Signaling Technology, 2272, used at 1:1000 for western blots), mouse anti-Myc (Cell Signaling Technology, 2276, used at 5 μg/ml for co-immunoprecipitation), rabbit anti-FLAG (Cell Signaling Technology, 2368, used for co-immunoprecipitation experiments involving C3 transferase at 1:1000), rabbit anti-GFP (Cell Signaling Technology, 2555, used for co-immunoprecipitation experiments involving C3 transferase at 1:1000), rabbit anti-GFP (Thermo Fisher Scientific, A-11122, used at 1:1000 for western blot), phospho-ERK1/2 (Cell Signaling Technology, 9101, used at 1:1000 for western blot), rabbit anti-RhoA (Cell Signaling Technology, 2117, used at 1:1000 for western blot), rabbit anti-RhoB (Cell Signaling Technology, 2098, used at 1:1000 for western blot), rabbit anti-RhoC (Cell Signaling Technology, 3430, used at 1:1000 for western blot) Secondary antibodies used were HRP-conjugated monoclonal mouse anti-rabbit IgG, light chain specific (Jackson ImmuonoResearch, 211-032-171, clone 5A6-1D10, used at 1:100,000) and goat anti-rabbit IgG (Li-COR, 926-32211, used at 1:50,000)

Validation

For all antibodies from Cell Signaling: Examination of several cell lines and/or tissues of known expression levels allows accurate determination of species cross-reactivity and verifies specificity. The use of siRNA transfection or knockout cell lines verifies target specificity. Side-by-side comparison of lots to ensure lot-to-lot consistency. Optimal dilutions and buffers are predetermined, positive and negative cell extracts are specified, and detailed protocols are already optimized, saving valuable time and reagents.

GFP (Thermo Fisher Scientific, A-11122): This antibody has been used in over 1,600 papers as listed on the product website (https://www.thermofisher.com/antibody/product/GFP-Antibody-Polyclonal/A-11122). The antibody to GFP was further validated by our lab by western blot and immunofluorescence using untransfected and epitope-tag transfected cells as negative and positive controls, respectively.

HRP conjugated secondary: From manufacturer: The antibody has been tested by ELISA to ensure minimal cross-reaction with bovine, goat, armenian hamster, horse, human, mouse, rat and sheep immunoglobulins, but it may cross-react with immunoglobulins from other species.

Eukaryotic cell lines

Policy information about cell lines and Sex and Gender in Research

Cell line source(s)

Human embryonic kidney (HEK) 293S GnTI- cells are purchased from ATCC, X. laevis oocytes are purchased from Ecocyte (Autsin, Texas, USA), MN-1 cells were obtained from Dr. Kenneth Fischbeck at the NIH. HEK293T cell were obtained from ATCC (CRL-11268). There is no commercial source for MN-1 cells.

Authentication

Cell lines were not authenticated.

Mycoplasma contamination

Cell lines were not tested for mycoplasma contamination.

Commonly misidentified lines
(See ICLAC register)

No commonly misidentified cell line was used in this study.
